# Expression profile of translation initiation factor eIF2B5 in diffuse large B-cell lymphoma and its correlation to clinical outcome

**DOI:** 10.1038/s41408-018-0112-5

**Published:** 2018-08-22

**Authors:** Julia J. Unterluggauer, Katharina Prochazka, Peter V. Tomazic, Heinrich J. Huber, Rita Seeboeck, Karoline Fechter, Elisabeth Steinbauer, Verena Gruber, Julia Feichtinger, Martin Pichler, Marc A. Weniger, Ralf Küppers, Heinz Sill, Rudolf Schicho, Peter Neumeister, Christine Beham-Schmid, Alexander J. A. Deutsch, Johannes Haybaeck

**Affiliations:** 1 Diagnostic and Research Institute of Pathology, Diagnostic and Research Center for Molecular BioMedicine, Medical University of Graz, Graz, Austria; 2 Division of Hematology, Medical University of Graz, Graz, Austria; 3 Department of Otorhinolaryngology, Medical University of Graz, Graz, Austria; 4 Institute for Automation Engineering, Otto von Guericke University Magdeburg, Magdeburg, Germany; 5 Department Life Sciences, IMC University of Applied Sciences Krems, Krems, Austria; 6 Institute of Computational Biotechnology, Graz University of Technology, Graz, Austria; 7 BioTechMed Omics Center Graz, Graz, Austria; 8 Department of Internal Medicine, Medical University of Graz, Graz, Austria; 9 Institute of Cell Biology (Cancer Research), University of Duisburg-Essen, Essen, Germany; 10 German Cancer Consortium (DKTK), Heidelberg, Germany; 11 Division of Pharmacology, Otto Loewi Research Center, Medical University of Graz, Graz, Austria; 12 Department of Pathology, Medical Faculty, Otto von Guericke University Magdeburg, Magdeburg, Germany

Diffuse large B-cell lymphoma (DLBCL) is the most frequent subtype of B-cell non-Hodgkin lymphoma in adults. The disease is curable in a proportion of patients using chemo-immunotherapy. Nevertheless, 40% of patients still succumb to this B-cell malignancy. One reason for this lack of success in curing all patients is the heterogeneity of the disease^1^.

Cellular protein synthesis starts in the translation initiation phase. The translation initiation process is characterized by the formation of an elongation-competent 80S ribosome, in which the joining of the initiator transfer RNA (tRNA) with the start codon is realized. For this, eukaryotic initiation factors (eIFs) are crucial^2^. Importantly, overexpression of eIF-subunits has been demonstrated in various cancer entities so far^3^. Moreover, the last two decades have also seen several studies on the role of different eIF-subunits in DLBCL, focusing particularly on eIF4-subunits and suggesting also therapeutic relevance^4–7^. However, apart from studies on some specific eIF-subunits, a large number of eIF-subunits has not been investigated in DLBCL until now.

To close this gap, we aimed to exploratively analyze a great range of eIF-subunits for their relevance in DLBCL pathogenesis. By quantitative real-time PCR (qRT-PCR) we thereby analyzed a set of 16 eIF-subunits in a cohort of primary (*n* = 34) and secondary (*n* = 22) DLBCL patients, the latter originating from a pre-existing follicular lymphoma (FL) grade 3 (methods and patient characteristics are described in detail in Supplementary information with Supplementary Material and Methods and Supplementary Tables S1 and S2). Germinal center B-cell specimens, isolated from non-neoplastic tonsils of five patients undergoing routine tonsillectomy, were included as controls. Remarkably, 12 out of 16 tested eIF-subunits, namely *EIF1, EIF1A, EIF2B3*, *EIF2B4*, *EIF2B5*, *EIF2S1*, *EIF3D*, *EIF4A2*, *EIF4E*, *EIF4EBP1*, *EIF4G2*, and *EIF5*, showed a higher expression in DLBCL as compared with non-neoplastic controls at the messenger RNA (mRNA) level (Supplementary Figures S1 and S2, Fig. 1a, Fig. 2a, *p* < 0.003). *EIF1A*, *EIF3D*, and *EIF2B5* even showed an 8- to 40-fold higher expression in DLBCL when compared with normal germinal center B cells (Fig. 1a, Fig. 2a, *p* < 0.001). To confirm the mRNA expression at the protein level, we performed immunohistochemistry (IHC) for the respective gene products eIF1A, eIF2B5 and eIF3d on primary and secondary DLBCL specimens (*n* = 22 for eIF1A and eIF3d, *n* = 49 for eIF2B5). Expression of these factors by DLBCL cells was thereby compared with germinal center centroblasts of non-neoplastic tonsils (*n* = 10). Indeed, higher scores in DLBCL cells confirmed our mRNA data (Fig. 1b, c, Supplementary Table S3, *p* = 0.048 for eIF1A, *p* = 0.015 for eIF3d; Fig. 2b, c, Supplementary Table S4, *p* < 0.001 for eIF2B5). Most importantly, all three aforementioned eIF-subunits have not been previously analyzed in DLBCL. eIF1A is a translation initiation factor involved in start codon screening^3^. eIF3d is one of the 13 subunits of the eIF3-complex, which not only promotes tRNA and mRNA recruitment to the ribosome but also start codon scanning^2^. Notably, eIF3d recently was found to enable eIF4E-independent mRNA recruitment to the ribosome and to allow for specialized translation of mRNAs controlling cell growth, thereby assigning eIF3d a key role within the eIF3-complex^8,9^. Owing to this important function, our findings suggest that eIF3d might be implicated in the increased proliferation of DLBCL. eIF2B5 (also known as eIF2Bε) is one of the five subunits of the eIF2B-complex, which is necessary for recycling of eIF2-GTP to enable recruitment of the first tRNA to the ribosome^10^. Gene mutations in any of the five subunits have been linked to leukoencephalopathy with vanishing white matter^11^. However, nothing has been reported so far regarding a possible disease-relevant role of eIF2B5 in DLBCL.

**Fig. 1:**
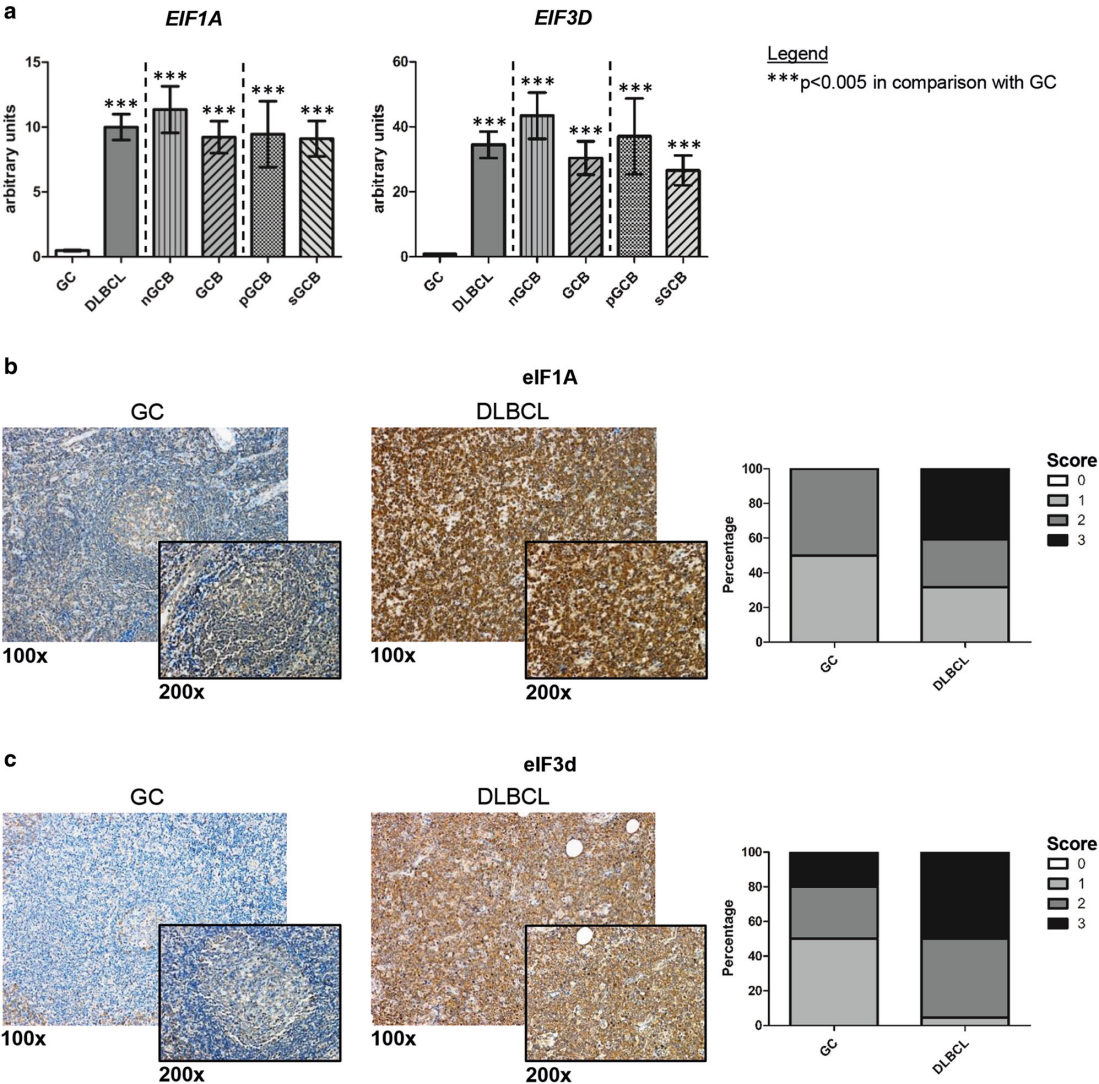
Analysis of *EIF1A*/eIF1A and *EIF3D*/eIF3d expression in DLBCL and non-neoplastic B-cell controls. Comparison of subtype-specific mRNA expression of *EIF1A* and *EIF3D* in DLBCL and non-neoplastic germinal center B cells determined by qRT-PCR **a**. The figures depict relative mRNA levels. Each bar represents the mean value ± standard error of the mean. Non-neoplastic germinal center B cells (GC) were compared with DLBCL, split up into the subtypes nGCB-DLBCL (nGCB) and GCB-DLBCL (GCB). Within the GCB-DLBCL subtype, primary GCB-DLBCL (pGCB) and secondary GCB-DLBCL originating from a FL grade 3 (sGCB) were distinguished. Representative immunohistochemical staining examples (× 100 and × 200 magnification) of **b**. eIF1A and **c**. eIF3d expression in tonsillar germinal centers (GC) and DLBCL specimens, as well as graphical assessment of staining intensities. All images were captured by using an Olympus BX51 microscope and an Olympus E-330 camera. Immunohistochemical staining intensity of DLBCL cells was compared with that of non-neoplastic tonsillar germinal center centroblasts (GC bar)

**Fig. 2:**
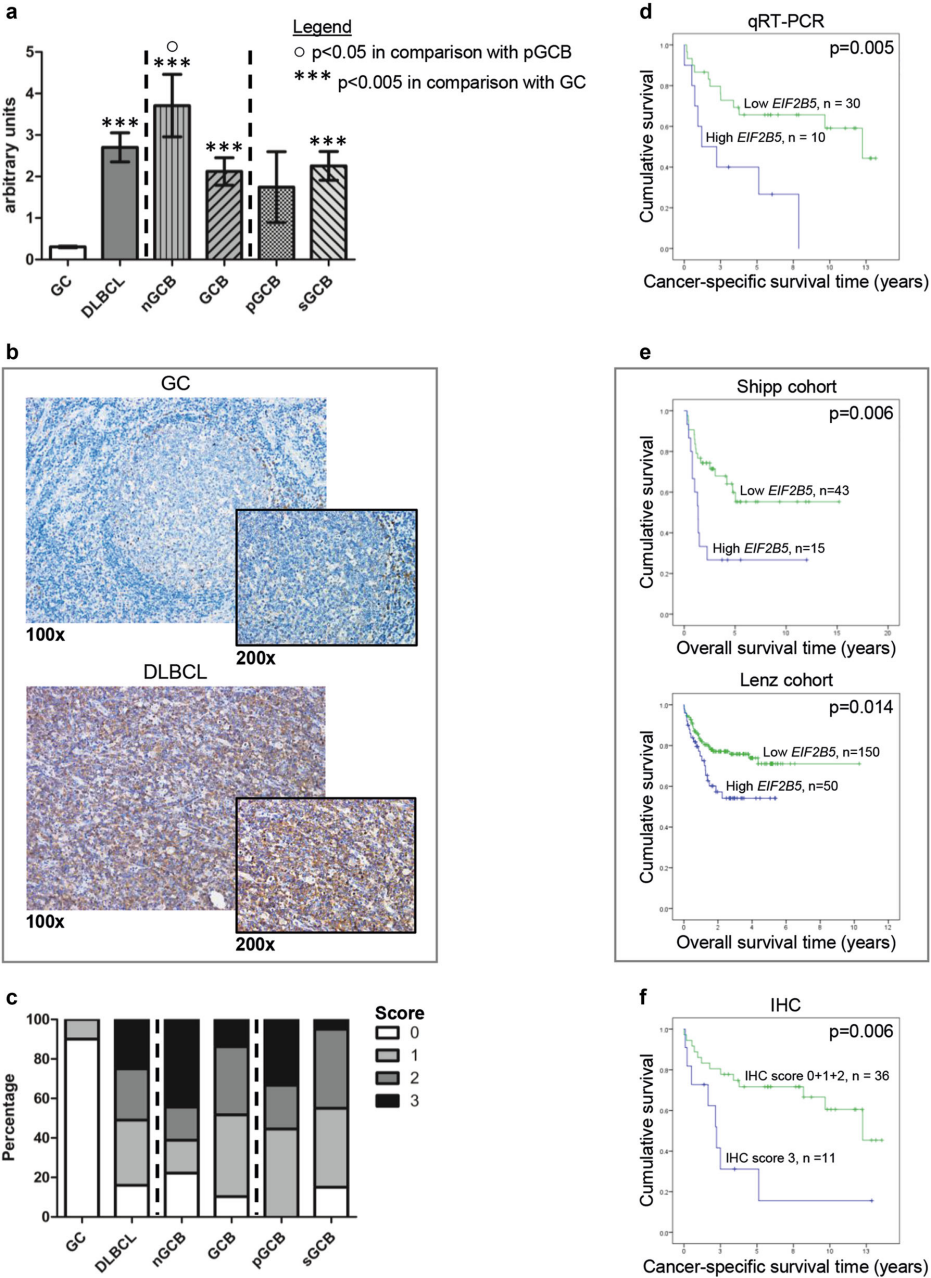
Analysis of *EIF2B5*/eIF2B5 expression in DLBCL and non-neoplastic B-cell controls. Comparison of subtype-specific *EIF2B5* mRNA expression in DLBCL and non-neoplastic germinal center B cells determined by qRT-PCR **a**. The subtype-specific mRNA expression analysis depicts relative mRNA levels. Each bar represents the mean value ± standard error of the mean. Non-neoplastic germinal center B cells (GC) were compared with DLBCL, split up into the subtypes nGCB-DLBCL (nGCB) and GCB-DLBCL (GCB). Within the GCB-DLBCL subtype, primary GCB-DLBCL (pGCB) and secondary GCB-DLBCL originating from a FL grade 3 (sGCB) were distinguished. Representative immunohistochemical staining examples (× 100 and × 200 magnification) of eIF2B5 expression in tonsillar germinal centers (GC) and DLBCL specimens **b**. All images were captured by using an Olympus BX51 microscope and an Olympus E-330 camera. Graphical assessment of immunohistochemical staining intensities **c**. For abbreviations see **a**. Immunohistochemical staining intensity of DLBCL cells was compared with that of non-neoplastic tonsillar germinal center centroblasts (GC bar). Patient survival in relation to *EIF2B5* mRNA levels in the study cohort determined by qRT-PCR **d**. Patient survival in relation to *EIF2B5* mRNA levels in the publicly available Shipp^13^ and Lenz^14^ data sets **e**. Patients with lower *EIF2B5* mRNA expression than the 3rd quartile of *EIF2B5* expression levels of each cohort are shown in green and those with higher expression in blue. **f** Patient survival in relation to IHC data on eIF2B5 expression in the study cohort

In addition, we compared in an explorative manner at the mRNA level the two disease-relevant subtypes of DLBCL using the Hans classification^12^ in our cohort. Secondary DLBCL originating from FL grade 3 was added to the germinal center B-cell-like (GCB)-DLBCL subtype, see also Supplementary information. Eight of the 16 tested eIF-subunits showed considerably higher expression in the non-GCB (nGCB)-DLBCL subtype compared with the GCB-DLBCL subtype: *EIF1*, *EIF2A*, *EIF2S1*, *EIF2B3*, *EIF2B4*, *EIF2B5*, *EIF4E*, and *EIF5* (Supplementary Figures S1 and S2, Fig. 2a, in comparison with both GCB-DLBCL subgroups and primary/secondary GCB-DLBCLs alone: *p* < 0.05). Taking the mRNA analysis as a whole, our study thereby confirms results of previous studies by demonstrating higher *EIF4E* and *EIF4EBP1* expression in DLBCL and higher *EIF4E* expression in the more aggressive nGCB-DLBCL subtype, what is consistent with the study of Wang et al. that shows a link between higher eIF4e expression and lymphoma aggressiveness.^4–6^ *EIF2B5* and *EIF5* exhibited a twofold and 1.5-fold higher mRNA expression in the nGCB-DLBCLs of our study cohort, respectively (Fig. 2a, *p* = 0.058 in comparison with both GCB-DLBCL subgroups, *p* = 0.043 in comparison with primary GCB-DLBCL alone for *EIF2B5*; Supplementary Figure S1, *p* = 0.007 in comparison with both GCB-DLBCL subgroups, *p* = 0.001 in comparison with primary GCB-DLBCL alone for *EIF5*). To confirm this differential expression, we analyzed eIF2B5 protein expression by IHC staining in the DLBCL patient specimens of our cohort, which could be classified regarding DLBCL subtype (nGCB-DLBCL *n* = 18, primary GCB-DLBCL *n* = 9, secondary GCB-DLBCL *n* = 20). Importantly, also IHC analysis revealed a more intense staining pattern of eIF2B5 in nGCB-DLBCL (*p* = 0.038 in comparison with both GCB-DLBCL subgroups), confirming our mRNA data and strengthening the role of eIF2B5 in DLBCL pathogenesis (Fig. 2c, Supplementary Table S4).

To further investigate the relevance of eIFs in lymphomagenesis and to analyze their prognostic properties, we exploratively set their expression in relation to cancer-specific survival in our lymphoma cohort. By dividing the patients of our lymphoma cohort into two groups using the 3rd quartile of the mRNA expression levels for the given eIF-subunit, we detected that high expression of the eIF-subunits *EIF2B5*, *EIF4E*, and *EIF5* correlated with worse patient outcome (Fig. 2d, for *EIF2B5* *p* = 0.005; Supplementary Figure S3, for *EIF4E* *p* = 0.017, and for *EIF5* *p* = 0.001). To validate our findings, we used two publicly available mRNA expression data sets published by Shipp et al.^13^ and Lenz et al.^14^, that include 58 and 200 DLBCL patients, respectively. Like in our patient cohort, in both external patient cohorts pretreatment samples were used to study patient survival. Comparing cohort characteristics, the Shipp and the Lenz cohort, in general, showed lower International Prognostic Index scores than our study cohort. In contrast, Ann Arbor Stage and DLBCL subtype distribution, which were only reported in the Lenz cohort, were similar to the ones of our study cohort, whereas patients of the Lenz cohort were in general of younger age (Supplementary Table S5). Importantly, when analyzing patient survival in the Shipp and Lenz data sets in relation to eIF levels, it was again *EIF2B5* whose expression could be confirmed in both external data sets regarding its prognostic potential (Fig. 2e, *p* = 0.006 and *p* = 0.014, respectively). Furthermore, analyzing the IHC data of eIF2B5 in our lymphoma cohort regarding patient outcome revealed that a very strong eIF2B5 expression (the maximum score 3—seen in approximately one quarter of all patients) was again associated with a significantly worse outcome (Fig. 2f, *p* = 0.006). This demonstrates the promising prognostic potential of eIF2B5 also at the protein level, which reflects the effector state of the mRNA. In contrast to qRT-PCR, IHC is a method frequently used in routine pathology and could thereby allow for analysis of eIF2B5 in daily clinical practice to stratify patients risk a priori.

To further investigate the prognostic relevance of *EIF2B5*/eIF2B5 in our study cohort, we tested the *EIF2B5* mRNA data in a multivariate setting by using the Cox proportional hazards model adjusting for the covariates “age”, “stage”, “sex”, and “subtype” (Supplementary Table S6). The multivariate analysis revealed that high *EIF2B5* expression (*p* = 0.003, hazard ratio of 5.615), together with advanced stage (*p* = 0.006, hazard ratio of 7.121) and presence of nGCB-DLBCL subtype (*p* = 0.002, hazard ratio of 7.422), was associated with worse patient outcome independent of the other tested covariates. This additionally underlines the importance of *EIF2B5* expression in DLBCL pathogenesis. Prognostic utility of *EIF2B5* has already been shown, e.g., in ovarian cancer, where rare alleles of *EIF2B5* were found to be linked to improved survival^15^. Our data suggest that *EIF2B5*/eIF2B5 might serve as prognostic marker in DLBCL as well.

To conclude, our study on DLBCL reveals an important role of a great range of so far untested eIF-subunits, demonstrating overexpression of eIF-subunits in DLBCL and higher expression in the more aggressive nGCB-DLBCL subtype. Though, it has to be mentioned that regarding evaluation of clinical utility the relatively small size of the patient cohort represents an important limitation of our study. Nevertheless, for eIF1A, eIF3d, and eIF2B5, we demonstrate at the mRNA as well as protein level for the first time that these factors potentially possess a disease-relevant role in DLBCL. It is especially eIF2B5 that reveals a so far undiscovered potential in DLBCL research, as our results suggest possible therapeutic and prognostic utility of this eIF-subunit in DLBCL pathogenesis.

## Electronic supplementary material

Supplementary Figure S1

Supplementary Figure S2

Supplementary Figure S3

Supplementary information
