# Supplementary figures and images for: Expression profile of translation initiation factor eIF2B5 in diffuse large B-cell lymphoma and its correlation to clinical outcome
Source: Blood Cancer J. 2018 Aug 22;8(9):79. doi: 10.1038/s41408-018-0112-5 (PMC6127263; doi:10.1038/s41408-018-0112-5)

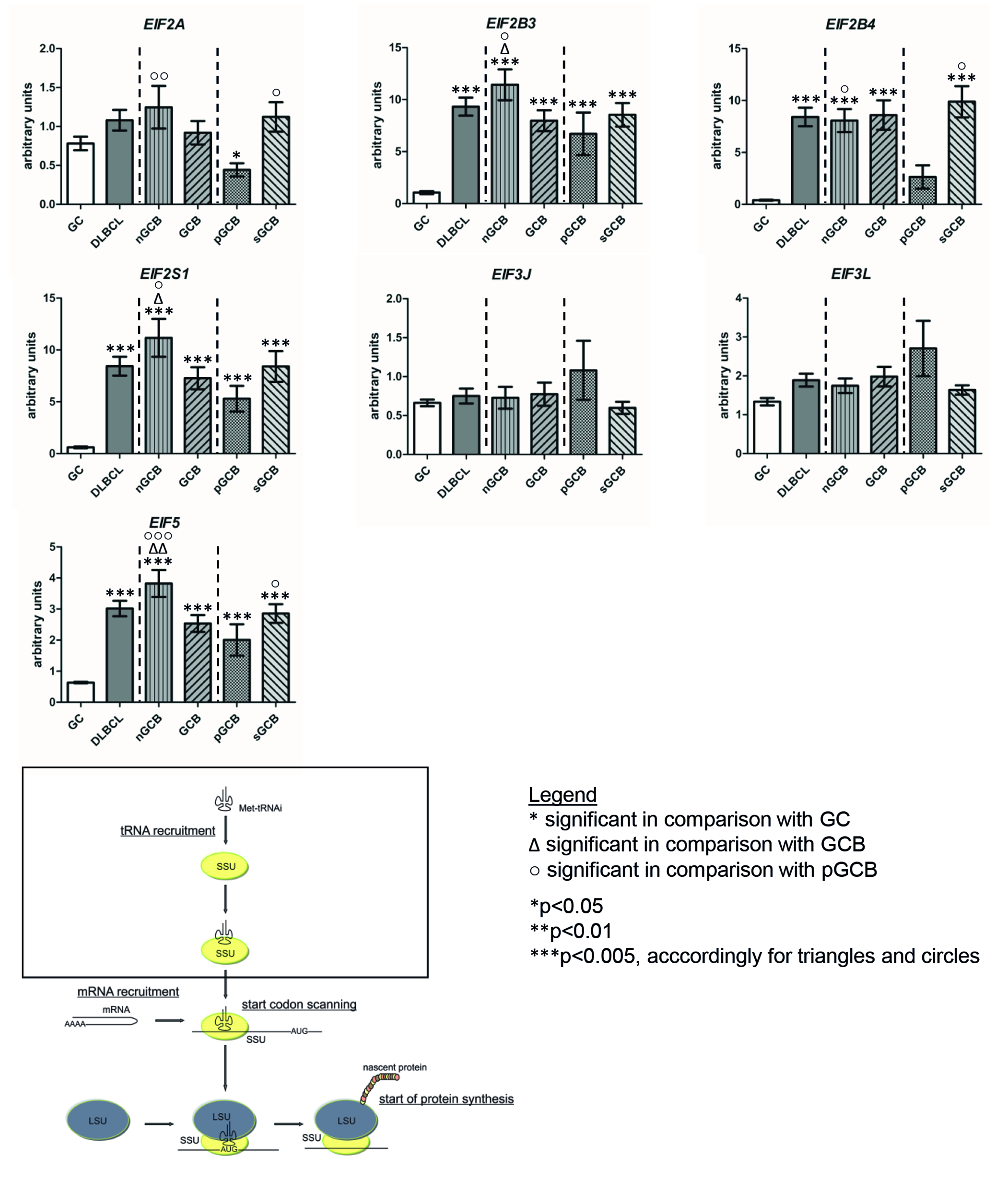

Supplement: Supplementary file 1 — Supplementary Figure S1 [file 41408_2018_112_MOESM1_ESM.tif]

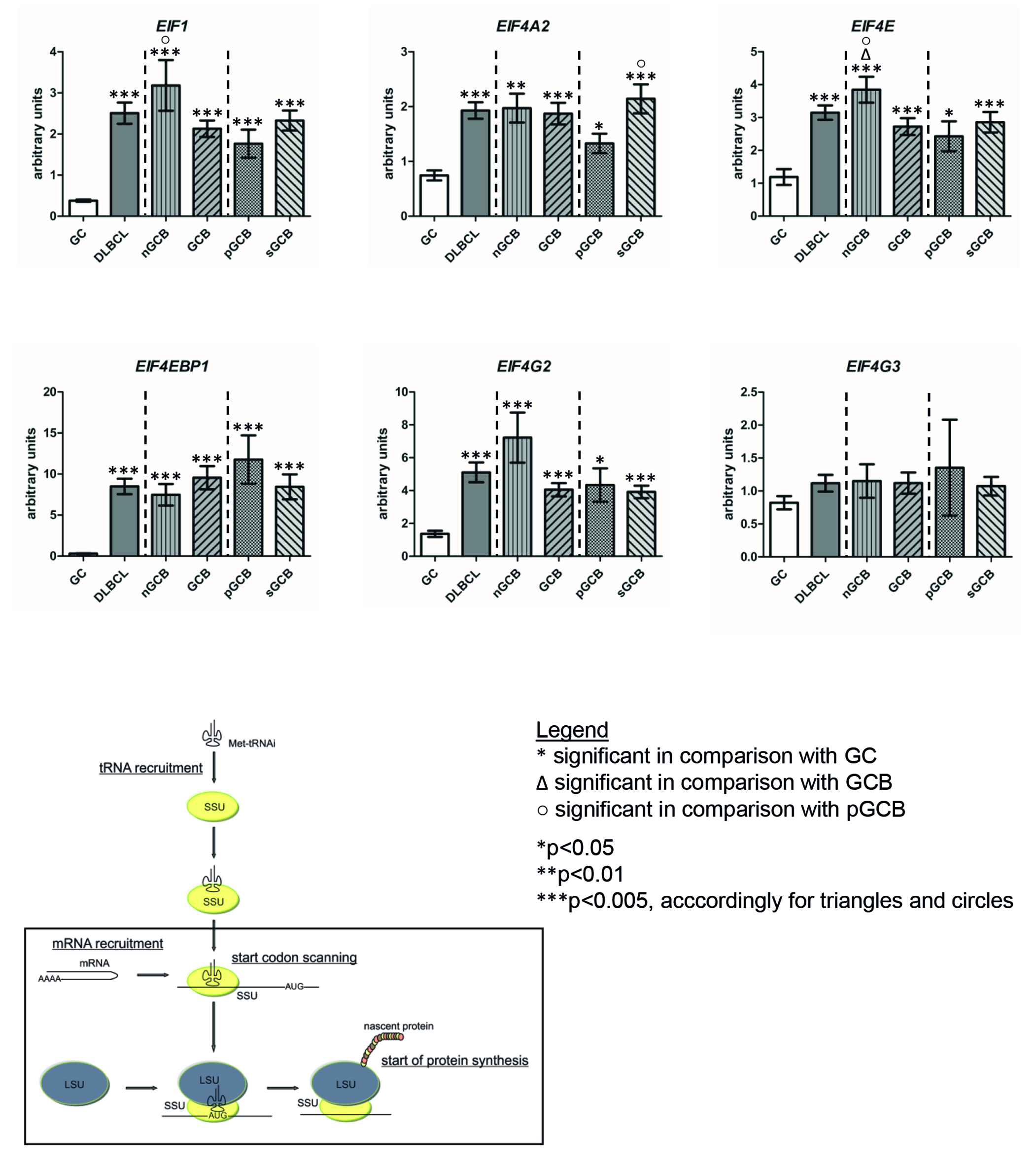

Supplement: Supplementary file 2 — Supplementary Figure S2 [file 41408_2018_112_MOESM2_ESM.tif]

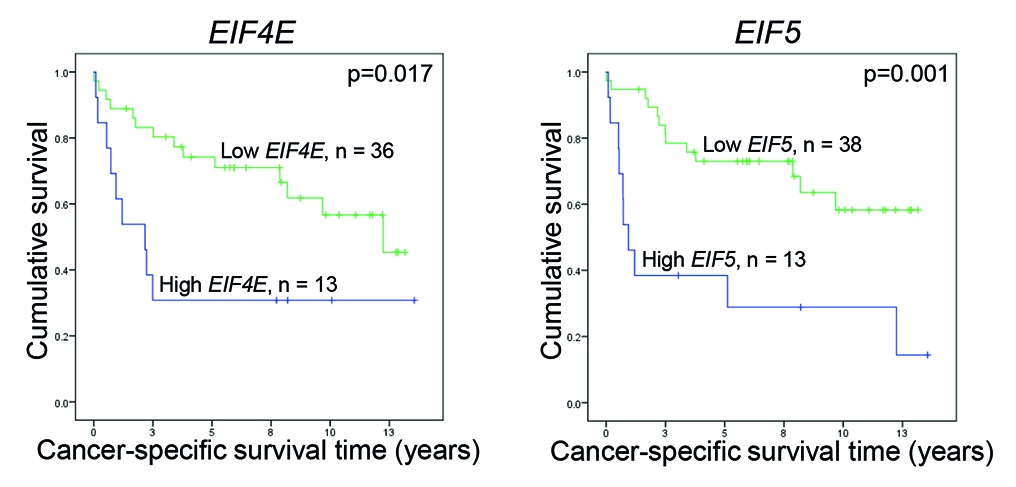

Supplement: Supplementary file 3 — Supplementary Figure S3 [file 41408_2018_112_MOESM3_ESM.tif]
