# Supplementary material for: Expression profile of translation initiation factor eIF2B5 in diffuse large B-cell lymphoma and its correlation to clinical outcome
Source: Blood Cancer J. 2018 Aug 22;8(9):79. doi: 10.1038/s41408-018-0112-5 (PMC6127263; doi:10.1038/s41408-018-0112-5)
Supplement: Supplementary file 4 — Supplementary information [file 41408_2018_112_MOESM4_ESM.pdf]

Supplementary Material and Methods

Patient specimens and non-neoplastic controls

Pseudonymized tumor samples of 56 DLBCL patients were collected at the Institute of Pathology of the Medical University of Graz between 2002 and 2010. Patients received R-CHOP (i.e., rituximab plus combination chemotherapy with cyclophosphamide, doxorubicin, vincristine, and prednisone)-like regimens, and were followed-up until 2016, with a median follow-up time of 5.7 years. Following the WHO classification[1], 22 of these tissue specimens were obtained from secondary DLBCL arising from FL grade 3, and 34 were obtained from primary DLBCL. Using the Hans algorithm[2] for IHC profiles, 12 primary DLBCL tumor samples were classified as GCB-DLBCL, 19 as nGCB-DLBCL, and 3 were not classifiable. For these 3 cases, re-classification was not possible due to lack of tumor material. Secondary DLBCL originating from FL exhibits an expression pattern similar to that of GCB-DLBCL[3]. Therefore, these cases were included into this subtype for all analyses (**Supplementary Table S1**). Only fresh frozen material was used for qRT-PCR approaches, and paraffin-embedded material was used for IHC. For this retrospective study, we used patient specimens collected for routine diagnostic investigations. Therefore, no written informed consent from patients was obtained, which was approved by the ethics committee of the Medical University of Graz (Number of ethical application: 28-040 ex 15/16). Non-neoplastic germinal center B cells ($CD20^{high}$ and $CD38^{+}$) for qRT-PCR approaches were isolated using a FACS-ARIA3 cell sorter from five human tonsils collected from children and adolescents undergoing routine tonsillectomy with informed consent, as approved by the local ethics committee of the Medical School of the University Duisburg-Essen (Number of ethical application: 11-4799-30). For that purpose, the following antibodies were used: anti-CD20-antibody (purchased from Becton Dickinson, Franklin Lakes, NJ, USA; order number: 562873) and anti-CD38-antibody (purchased from Becton Dickinson; order number: 551400). For immunohistochemical analysis and qRT-PCR calibration, human tonsils were collected from patients undergoing routine tonsillectomy with informed consent, as approved by the local ethics committee of the Medical University of Graz.

RNA isolation and mRNA expression analysis

RNA was extracted from fresh frozen DLBCL patient tissues and non-neoplastic germinal center B cells using miRNeasy Mini Kit (Qiagen, Hilden, Germany) according to the manufacturer's instructions. The isolated RNA was then transcribed into cDNA using the High Capacity cDNA Reverse Transcription Kit (Applied Biosystems, Foster City, CA, USA) according to the manufacturer's instructions.

qRT-PCR was performed in triplicate using a C1000 Touch Thermal Cycler CFX 384 Real-Time System (Bio-Rad Laboratories, Inc., Hercules, CA, USA). Reaction mix: 1x of GoTaq qPCR Master Mix (Promega, Madison, WI, USA), 0.1 µM of forward and reverse primer for respective gene detection, 1:50 diluted cDNA. Cycling protocol: Hot-start activation (95°C for 2 min), 40 cycles of denaturation and annealing/extension (95°C for 15 s, 60°C for 30 s), followed by a dissociation phase (60-95°C). The primer pairs used are listed in **Supplementary Table S2**. Actin and GAPDH, known to exhibit one of the lowest variabilities among lymphoid malignancies[4], were used as housekeeping genes. Tonsillar cDNA served as calibrator. The results are expressed as relative units based on calculation $2^{-\Delta\Delta CT}$, which gives the relative amount of the target gene normalized to the endogenous control (geometric mean of the two housekeeping genes)[5]. The qRT-PCR results for each eIF-subunit were evaluated regarding the shape of amplification curves, standard deviation of triplicate C(t) values and unusual melting patterns (e.g., double peak). Specimens that did not fulfill these pre-established criteria were excluded from analysis.

IHC analysis

GCB- and nGCB-DLBCL classification was performed using IHC on paraffin-embedded material from the study patients. For expression analysis of eIF1A and eIF3d, we analyzed 10 non-neoplastic tonsils as well as 22 primary and secondary DLBCL specimens by IHC. For expression analysis of eIF2B5, we investigated 10 non-neoplastic tonsils as well as 49 primary and secondary DLBCL specimens. Formalin-fixed, paraffin-embedded tissue was pretreated in a water bath with Target Retrieval Solution (1:10, Dako, Glostrup, Denmark) for 40 min. Then the slides were incubated with the respective primary antibodies for 30 min at room temperature: anti-CD10-antibody (1:6, purchased

from Novocastra, Leica Biosystems, Wetzlar, Germany; order number: NCL-CD10-270), anti-MUM1-antibody (1:50, purchased from Dako; order number: M7259), anti-BCL6-antibody (1:100, purchased from Cell Marque, Rocklin, CA, USA; order number: 227M-96), anti-eIF1A-antibody (1:150, purchased from Abcam, Cambridge, United Kingdom; order number: ab177939), anti-eIF2B5-antibody (1:20, purchased from Santa Cruz Biotechnology, Dallas, TX, USA; order number: sc-55558) and anti-eIF3d-antibody (1:25, purchased from Santa Cruz Biotechnology; order number: sc-28856). In the case of anti-BCL6-antibody, the slides were pretreated with proteinase K (1:1500, Dako) for 10 min before primary antibody incubation. For staining, kit K5001 (Dako) and the automated stainer intelliPATH FLX® (Biocare Medical, Pacheco, CA, USA) were used according to the manufacturer's instructions. We included tissues known to contain the respective antigens as controls (positive controls). Replacing the primary antibody with normal serum always produced negative results (negative controls).

DLBCL specimens were investigated regarding the staining intensities of the DLBCL cells. In case of non-neoplastic tonsils, staining of the germinal center centroblasts served as a non-neoplastic control for DLBCL. Score 0 was assigned to no staining and scores 1, 2 and 3 to weak, moderate and strong staining intensity, respectively.

Statistical analysis

For statistical analysis, IBM SPSS Statistics for Windows, Version 23.0 (IBM Corp., Armonk, NY, USA) and GraphPad Prism Version 5.01 for Windows (GraphPad Software, Inc., La Jolla, CA, USA) were used. The Shapiro-Wilk test was used to test for normality of distribution. Depending on the test result, a t-test or a Mann-Whitney test, its non-parametric counterpart, was used to investigate mRNA expression for differences between germinal center B cells, DLBCL and the respective DLBCL subtypes in an explorative manner (two-sided p-value). In the corresponding graphs, mean values +/- standard error of the mean are depicted (see figure legends). To analyze differential protein expression measured by IHC, a Fisher's exact test was applied (two-sided p-value).

Survival analysis

To analyze the possible prognostic impact of eIF-subunit expression in an explorative manner, we integrated survival data of the investigated patient cohort (depicted in **Supplementary Table S1**, median follow-up time: 5.7 years) into our gene/protein expression analyses. Two patients were lost to follow-up and therefore had to be excluded generally from all analyses. Patient numbers are indicated in **Fig. 2**, **Supplementary Fig. S3** and **Supplementary Table S6**. Based on the mRNA data, patients were subdivided into two groups using the 3rd quartile of the respective eIF-subunit mRNA expression levels within the cohort (termed as "high eIF-subunit expression" and "low eIF-subunit expression"). Based on the IHC data, patients with a score of 3 were opposed to the rest. Univariate survival analysis was performed by IBM SPSS Statistics for Windows, Version 23.0 (IBM Corp.) using Kaplan-Meier plots to depict survival outcome and the log-rank test to statistically evaluate differential survival. Furthermore, we tested two unrelated patient cohorts: For this purpose, we used the mRNA expression data sets published by Shipp *et al.*[6] and Lenz *et al.*[7] (n=58 and n=200, respectively). Again, the patients were subdivided into two groups using the 3rd quartile of the mRNA expression levels.

To analyze cancer-specific survival in relation to *EIF2B5* levels, while adjusting for other potential nuisance factors (multivariate analysis), we used the Cox proportional hazards model to analyze mRNA data of the study cohort. We corrected for the covariates "age" (continuous), "stage" (stage 1+2 vs stage 3+4), "sex" (female vs male) and "subtype" (GCB- vs nGCB-DLBCL). Uni- and multivariate Cox proportional hazards analysis of the covariates was performed. To test the applicability of the Cox proportional hazards model, we checked for the proportional hazards assumption as well as for outliers and the linearity of the continuous covariate "age" by Schönfeld, Deviance and Martingale residuals. To perform the analysis, we used IBM SPSS Statistics for Windows, Version 23.0 (IBM Corp.) and R Version 3.4.1 (ref. 8) using the R-packages "survival" and "survminer". The output is depicted in hazard ratios as well as corresponding 95% confidence intervals and p-values (**Supplementary Table S6**). A p-value < 0.05 was considered as statistically significant.

Supplementary Tables

Supplementary Table S1: Clinicopathologic characteristics of the patients included in this study.

Clinicopathologic parameters	*Patients (n=56)*	*Proportion*
Gender		
Male	28	50%
Female	28	50%
Age at diagnosis (years)		
<65	*23*	*41%*
Male	*12*	*52%*
Female	*11*	*48%*
>65	*33*	*59%*
Male	*16*	*48%*
Female	*17*	*52%*
Ann Arbor Stage		
1	*9*	*16%*
2	*12*	*21%*
3	*18*	*32%*
4	*16*	*29%*
Not classifiable	*1*	*2%*
Immunophenotype of DLBCL (Hans algorithm)		
GCB	*34*	*61%*
pGCB	*12*	*35%*
sGCB	*22*	*65%*
nGCB	*19*	*34%*
Not classifiable	*3*	*5%*

Not classifiable: The respective parameter could not be determined in the indicated number of patients; The indicated proportion of a parameter is always referred to the total number of study patients (n=56). Abbreviations: GCB-DLBCL (GCB), primary GCB-DLBCL (pGCB), secondary GCB-DLBCL originating from a FL grade 3 (sGCB), nGCB-DLBCL (nGCB).

Supplementary Table S2: Oligonucleotide sequences of primers for qRT-PCR.

Gene - Primer ID	Sequence (5’->3’)
EIF1-f<br>*EIF1*-r	GAAACGGCAGGAAGACCCTTA<br>CGGATGCTCAATTACAGTACCAT
EIF1A-f<br>*EIF1A*-r	AACAGACGCAGGGGTAAGAAT<br>CCTGAGCATACTCCTGACCAT
EIF2A-f<br>*EIF2A*-r	GACCCCAACCATACAAGGTGG<br>TTCTCCATAGTAGGAAGCTCCTG
EIF2B3-f<br>*EIF2B3*-r	GTGGAGGATCTCGGATGACAG<br>GCTCAAGCAGGTTCAATGGGT
EIF2B4-f<br>*EIF2B4*-r	CAGAGAACTGCCAGAATCGGG<br>GTTCGGCCTTACTCCGACC
EIF2B5-f<br>*EIF2B5*-r	TTCTGGTGGCCGATAGCTTC<br>AGCTTTCCAGCAACAAAAGACA
EIF2S1-f<br>*EIF2S1*-r	TGGTGAATGTCAGATCCATTGC<br>TAGAACGGATACGCCTTCTGG
EIF3D-f<br>*EIF3D*-r	CAGCGGAATCGAATGAGATTTGC<br>GTTTGGCACTCTTAGGCAGGA
EIF3J-f<br>*EIF3J*-r	TGCCCACAATCCCTTGAACA<br>GGCAGCAAGATCCAAGTTCC
EIF3L-f<br>*EIF3L*-r	GGAGGAGATTGACTTTCTTCGTT<br>TTGGATTTGTCTACCAGGGAATG
EIF4A2-f<br>*EIF4A2*-r	GAAGCCTTCCGCTATTCAGCA<br>CTTGGGTCTCCTTGAACTCAATC
EIF4E-f<br>*EIF4E*-r	TGCGGCTGATCTCCAAGTTTG<br>CCCACATAGGCTCAATACCATC
EIF4EBP1-f<br>*EIF4EBP1*-r	CACCCCGGGAGGTACCAGGATC<br>CGCCCGCCCGCTTATCTTCT
EIF4G2-f<br>*EIF4G2*-r	AGGGCAAAACGCTCAGAAATG<br>TCCTGAAGATTGCATCATGTCG
EIF4G3-f<br>*EIF4G3*-r	CCTAGAGCTACCATCCCGAAC<br>GGGCCACTATGACGGTACTG
EIF5-f<br>*EIF5*-r	AGCGTGTCAGACCAGTTCTAT<br>CTGTCTTGATTCCATTGCCTTTG
ACTB-f<br>*ACTB*-r	CTGGAACGGTGAAGGTGACA<br>AAGGGACTTCCTGTAACAATGCA
GAPDH-f<br>*GAPDH*-r	AAGGTCGGAGTCAACGGATTT<br>ACCAGAGTTAAAAGCAGCCCTG

Supplementary Table S3: Immunohistochemical analysis of eIF1A and eIF3d in non-neoplastic tonsillar germinal center centroblasts (GC) and DLBCL specimens.

**eIF1A**	**0 (-)**	**1 (+)**	**2 (++)**	**3 (+++)**	**p-value**
GC	0/10	5/10	5/10	0/10	
DLBCL	0/22	7/22	6/22	9/22	0.048[a]
**eIF3d**					
GC	0/10	5/10	3/10	2/10	
DLBCL	0/22	1/22	10/22	11/22	0.015[a]

[a] $p<0.05$ vs GC

Supplementary Table S4: Immunohistochemical analysis of eIF2B5 in non-neoplastic tonsillar germinal center centroblasts (GC) and DLBCL specimens.

**eIF2B5**	**0 (-)**	**1 (+)**	**2 (++)**	**3 (+++)**	**p-value**
GC	9/10	1/10	0/10	0/10	
DLBCL					<0.001[a]
nGCB	4/18	3/18	3/18	8/18	0.003[a]
GCB (pGCB+sGCB)	3/29	12/29	10/29	4/29	<0.001[a], 0.038[b]
pGCB	0/9	4/9	2/9	3/9	<0.001[a]
sGCB	3/20	8/20	8/20	1/20	<0.001[a], 0.017[b]

Abbreviations: nGCB-DLBCL (nGCB), GCB-DLBCL (GCB), primary GCB-DLBCL (pGCB), secondary GCB-DLBCL originating from a FL grade 3 (sGCB).

[a]p<0.05 vs GC

[b]p<0.05 vs nGCB

Supplementary Table S5: Comparison of clinicopathologic characteristics of the patient cohort analyzed in this study and the Shipp[6] and the Lenz[7] patient cohort. In our study cohort the subtypes GCB-DLBCL and nGCB-DLBCL were distinguished. In contrast, in the Lenz data set the separation was performed between the subtypes GCB-DLBCL and activated B-cell-like-DLBCL. For ease of comparison, both differently determined GCB-DLBCL subtypes (GCB) as well as the nGCB-DLBCL subtype (nGCB) and activated B-cell-like-DLBCL subtype (ABC) are shown in one row. For each parameter, the patient number revealing the respective characteristic is shown, followed by the total patient number with information about this parameter and the resulting percentage of the parameter characteristic within the cohort (calculated out of the two aforementioned numbers). In contrast to both other patient cohorts, only the International Prognostic Index (IPI) was reported in the Shipp cohort, so that all other parameter classifications are not applicable (na).

Clinicopathologic parameters	***Study cohort***	***Shipp cohort***	***Lenz cohort***
Treatment regimen	R-CHOP (rituximab plus combination chemotherapy with cyclophosphamide, doxorubicin, vincristine, and prednisone)-like regimen	CHOP (cyclophosphamide, adriamycin, vincristine and prednisone)-like combination chemotherapy	R-CHOP (rituximab plus combination chemotherapy with cyclophosphamide, doxorubicin, vincristine, and prednisone)-like regimen
Total patient number	56	58	200
Age (years)			
>60	44/56 (79%)	na	109/200 (54%)
Ann Arbor Stage			
>2	34/55 (62%)	na	105/194 (54%)
DLBCL subtype			
GCB	34/53 (64%)	na	107/200 (54%)
nGCB / ABC	19/53 (36%)	na	93/200 (46%)
IPI (IPI score)			
Low (0-1)	15/54 (28%)	26/56 (46%)	62/156 (40%)
Intermediate (2-3)	27/54 (50%)	28/56 (50%)	73/156 (47%)
High (4-5)	12/54(22%)	2/56 (4%)	21/156 (13%)

Supplementary Table S6: Multivariate analysis using the Cox proportional hazards model to investigate the correlation between *EIF2B5* mRNA expression and cancer-specific survival in the study cohort. The indicated hazard ratio (HR) always defines the hazard ratio of the second group compared to the first and is depicted with its lower and upper 95% confidence interval (CI). Patients with lower *EIF2B5* mRNA expression than the 3rd quartile of *EIF2B5* expression levels of the cohort belong to the "low" expressing group and patients above the 3rd quartile belong to the "high" expressing group.

	**1st group**	**2nd group**	**HR**	**Lower 95% CI**	**Upper 95% CI**	**p-value**	
			**Univariate**				
**Sex**	female, n=27	male, n=27	1.674	0.732	3.832	0.222	
**Age**	continuous		1.054	1.012	1.098	**0.011**	*
**Stage**	1+2, n=20	3+4, n=34	2.104	0.824	5.371	0.120	
**Subtype**	GCB, n=32	nGCB, n=19	7.551	2.845	20.037	**<0.001**	***
EIF2B5	low, n=30	high, n=10	3.562	1.390	9.131	**0.008**	**
			**Multivariate**				
**Sex**	female, n=18	male, n=19	1.624	0.536	4.917	0.391	
**Age**	continuous		1.057	0.997	1.121	0.064	
**Stage**	1+2, n=11	3+4, n=26	7.121	1.778	28.520	**0.006**	**
**Subtype**	GCB, n=23	nGCB, n=14	7.422	2.073	26.576	**0.002**	***
EIF2B5	low, n=28	high, n=9	5.615	1.765	17.863	**0.003**	***

Abbreviations: GCB-DLBCL (GCB), nGCB-DLBCL (nGCB). *p<0.05, **p<0.01, ***p<0.005

Supplementary Figures

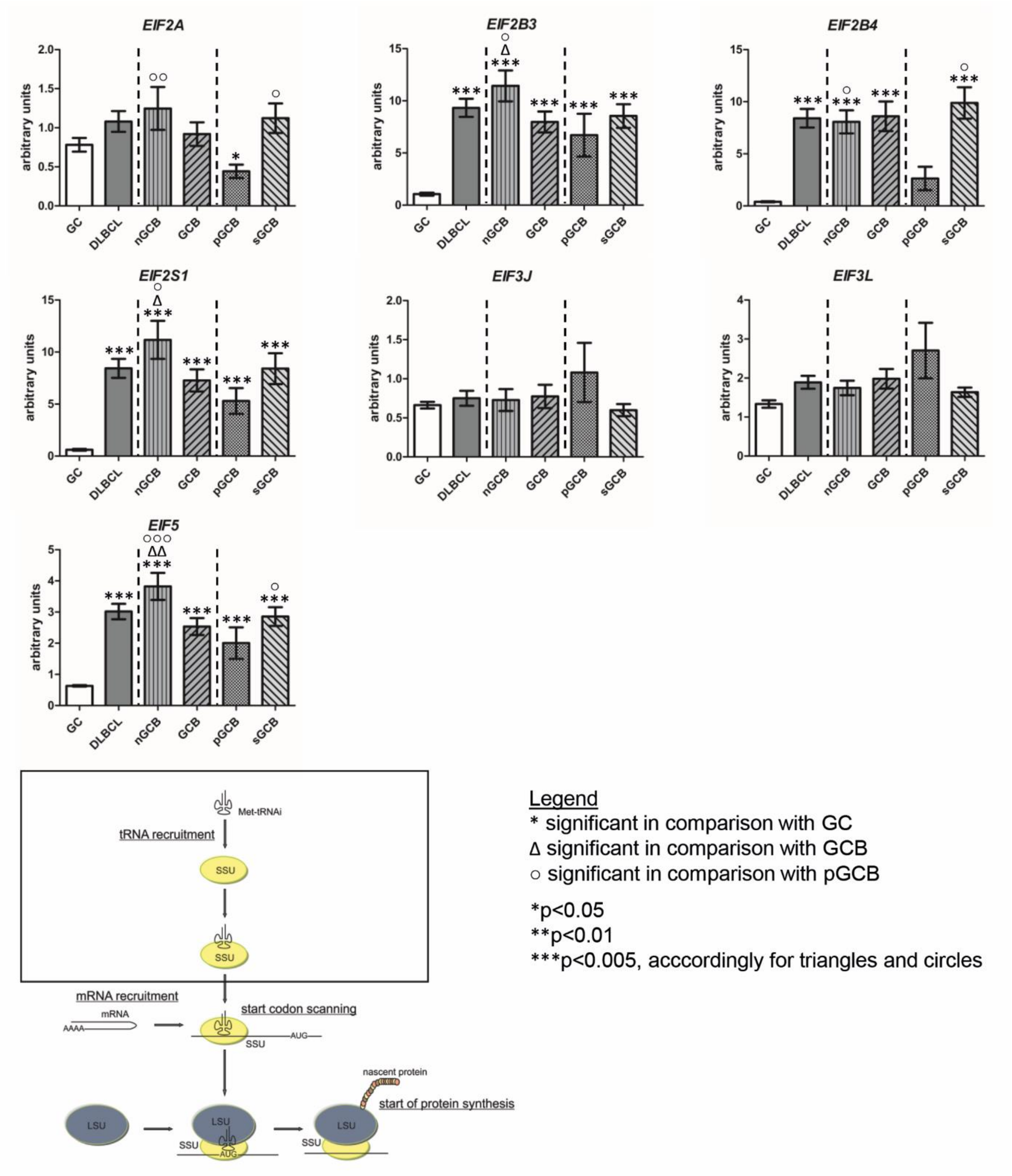

Supplementary Fig. S1: Comparison of subtype-specific mRNA expression of eIF-subunits in DLBCL and non-neoplastic germinal center B cells determined by qRT-PCR: eIFs involved at the first steps of translation initiation. This figure depicts relative mRNA levels. Each bar represents the mean value +/- standard error of the mean. Non-neoplastic germinal center B cells (GC) were compared with DLBCL, split up into the subtypes nGCB-DLBCL (nGCB) and GCB-DLBCL (GCB). Within the GCB-DLBCL subtype, primary GCB-DLBCL (pGCB) and secondary GCB-DLBCL originating from a FL grade 3 (sGCB) were distinguished. Start-tRNA (Met-tRNAi), large ribosomal subunit (LSU), small ribosomal subunit (SSU).

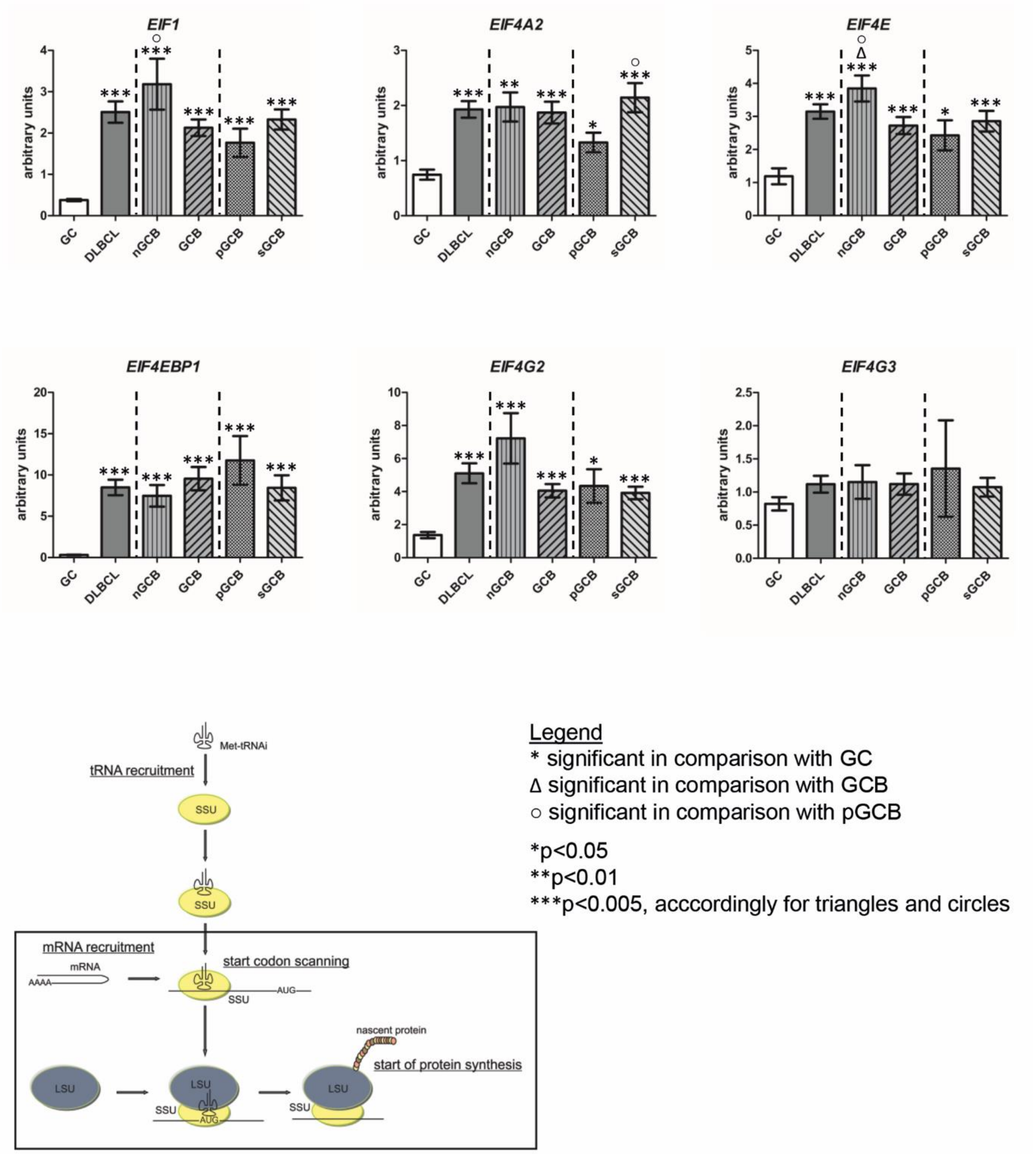

Supplementary Fig. S2: Comparison of subtype-specific mRNA expression of eIF-subunits in DLBCL and non-neoplastic germinal center B cells determined by qRT-PCR: eIFs involved in mRNA recruitment and start codon scanning. This figure depicts relative mRNA levels. Each bar represents the mean value +/- standard error of the mean. Non-neoplastic germinal center B cells (GC) were compared with DLBCL, split up into the subtypes nGCB-DLBCL (nGCB) and GCB-DLBCL (GCB). Within the GCB-DLBCL subtype, primary GCB-DLBCL (pGCB) and secondary GCB-DLBCL originating from a FL grade 3 (sGCB) were distinguished. Start-tRNA (Met-tRNAi), large ribosomal subunit (LSU), small ribosomal subunit (SSU).

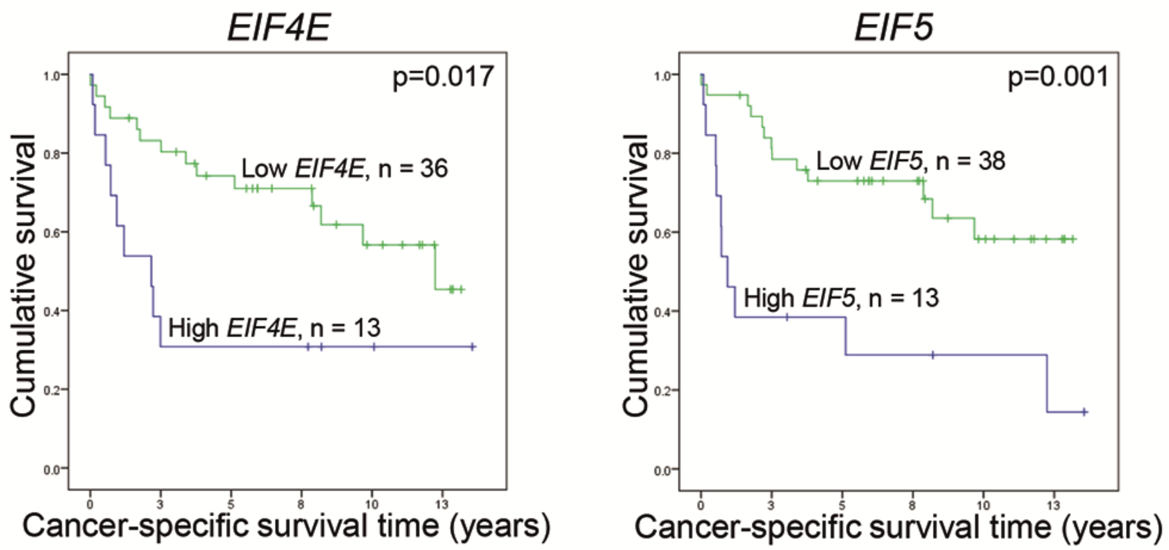

Supplementary Fig. S3: Patient survival in relation to *EIF4E* and *EIF5* mRNA levels in the study cohort determined by qRT-PCR. Patients with lower eIF-subunit mRNA expression than the 3rd quartile of the respective eIF-subunit expression levels of the cohort are shown in green and those with higher expression in blue.

References

1. Campo E, Swerdlow SH, Harris NL, Pileri S, Stein H, Jaffe ES. The 2008 WHO classification of lymphoid neoplasms and beyond: evolving concepts and practical applications. *Blood* 2011; **117**: 5019-5032.

2. Hans CP, Weisenburger DD, Greiner TC, Gascoyne RD, Delabie J, Ott G *et al.* Confirmation of the molecular classification of diffuse large B-cell lymphoma by immunohistochemistry using a tissue microarray. *Blood* 2004; **103**: 275-282.

3. Davies AJ, Rosenwald A, Wright G, Lee A, Last KW, Weisenburger DD *et al.* Transformation of follicular lymphoma to diffuse large B-cell lymphoma proceeds by distinct oncogenic mechanisms. *Br J Haematol* 2007; **136**: 286-293.

4. Lossos IS, Czerwinski DK, Wechser MA, Levy R. Optimization of quantitative real-time RT-PCR parameters for the study of lymphoid malignancies. *Leukemia* 2003; **17**: 789-795.

5. Vandesompele J, De Preter K, Pattyn F, Poppe B, Van Roy N, De Paepe A *et al.* Accurate normalization of real-time quantitative RT-PCR data by geometric averaging of multiple internal control genes. *Genome Biol* 2002; **3**: research0034.1-research0034.11.

6. Shipp MA, Ross KN, Tamayo P, Weng AP, Kutok JL, Aguiar RCT *et al.* Diffuse large B-cell lymphoma outcome prediction by gene-expression profiling and supervised machine learning. *Nat Med* 2002; **8**: 68-74.

7. Lenz G, Wright G, Dave SS, Xiao W, Powell J, Zhao H *et al.* Stromal Gene Signatures in Large-B-Cell Lymphomas. *N Engl J Med* 2008; **359**: 2313-2323.

8. R Development Core Team. R: A language and environment for statistical computing. *R Foundation for Statistical Computing*, Vienna, Austria; 2017.
